# T cell phenotypes associated with insulin resistance: results from the Berlin Aging Study II

**DOI:** 10.1186/s12979-020-00211-y

**Published:** 2020-12-21

**Authors:** Julia Sbierski-Kind, David Goldeck, Nikolaus Buchmann, Joachim Spranger, Hans-Dieter Volk, Elisabeth Steinhagen-Thiessen, Graham Pawelec, Ilja Demuth, Dominik Spira

**Affiliations:** 1 Charité-Universitätsmedizin Berlin, Corporate Member of Freie Universität Berlin, Humboldt-Universität zu Berlin, and Department of Endocrinology and Metabolism, Berlin Institute of Health, Chariteplatz 1, 10117 Berlin, Germany; 2 Present Address: Dept. of Laboratory Medicine, University of California, San Francisco, HSW1201U, Box 0451, 513 Parnassus Ave, San Francisco, CA 94143-0451 USA; 3 Fairfax Centre, Kidlington, UK; 4 Clinic for Cardiology, Charité Universitätsmedizin Berlin, Berlin, Germany; 5 Center for Cardiovascular Research (CCR), Department of Endocrinology and Metabolism, Charité-Universitätsmedizin Berlin, Berlin, Germany; 6 German Center for Cardiovascular Research (DZHK), partner site Berlin, Berlin, Germany; 7 Berlin Institute of Health (BIH) Center for Regenerative Therapies (BCRT), Charité - Universitätsmedizin Berlin, Berlin, Germany; 8 Berlin Center for Advanced Therapies (BeCAT), Charité - Universitätsmedizin Berlin, Berlin, Germany; 9 Institute of Medical Immunology, Charité - Universitätsmedizin Berlin, Berlin, Germany; 10 Department of Immunology, University of Tübingen, Tübingen, Germany; 11 Health Sciences North Research Institute, Sudbury, ON Canada

**Keywords:** Obesity, Insulin resistance, Systemic inflammation, Aging, T cell senescence

## Abstract

**Background:**

Obesity is associated with chronic low-grade inflammation leading to metabolic and cardiovascular diseases, but a subset of obese individuals is considered insulin sensitive (IS). The underlying pathophysiologic mechanisms remain elusive and clinical studies on the relationship between inflammatory markers and metabolically healthy obesity (MHO) are scarce.

**Methods:**

In this cross-sectional analysis, we included a sample of 437 older participants (60–84 years) from the Berlin Aging Study II (BASE-II). Peripheral blood mononuclear cells were isolated, immune cell subsets were analyzed with multiparameter flow cytometry and systemic cytokine levels were measured. Immune cell parameters were correlated with metabolic measures and multiple linear regression analysis was conducted and adjusted for various demographic and clinical factors.

**Results:**

We found that frequencies of naïve and memory CD4^+^ and CD8^+^ T cells inversely correlated with measures for insulin sensitivity in the older population. Moreover, the percentages of naïve CD4^+^ and CD8^+^ T cells were significantly higher, whereas activated T cells and IL-6 levels were lower in IS compared to insulin resistant (IR) obese individuals. The percentages of naïve CD4^+^ and CD8^+^ T cells were predictive for impaired insulin sensitivity (ß = 0.16, *p* = 0.01 and ß = 0.11, *p* = 0.04), and the association of naïve CD4^+^ T cells with insulin sensitivity persisted after multivariate adjustment (ß = 0.14, *p* = 0.02).

**Conclusions:**

These findings support the hypothesis that parameters of systemic inflammation can differentiate IS from IR obese individuals that are at higher risk for cardiometabolic diseases and may have clinical implications with regard to obesity treatment stratification.

**Trial registration:**

DRKS00009277. Registered 31 August 2015 - Retrospectively registered.

**Supplementary Information:**

The online version contains supplementary material available at 10.1186/s12979-020-00211-y.

## Background

Obesity, defined by the accumulation of abnormal or excess body fat, is a worldwide epidemic, with increasing prevalence in developed and developing countries and with a serious impact on human health. Worldwide, over 2 billion people are overweight or obese [1]. Obesity is a major risk factor for metabolic diseases, such as type 2 diabetes, dyslipidemia, fatty liver disease and cardiovascular diseases, such as hypertension, peripheral artery disease, myocardial infarction, stroke, and cancer [2–5]. .However, the large variation in the individual risk to develop insulin resistance or other obesity-related comorbidities has led to the concept of MHO [6]. Whereas the majority of obese people develop insulin resistance, about 20% are considered metabolically healthy with an age- and gender-dependent prevalence, and, thus, seem to be protected from insulin resistance [7]. The diagnosis of obesity and treatment options are based on the body mass index (BMI), which fails to reliably predict the predisposition to cardiometabolic diseases [8, 9]. Individuals with MHO have been defined by the absence of metabolic impairments or cardiovascular diseases, due to the lack of standardized MHO criteria. However, most recently the BioShare-EU project has proposed a unified definition of MHO, comparing clinical and metabolic factors of 10 population-based cohort studies from 7 countries, including fasting blood glucose [10, 11]. To guide personalized and risk-stratified obesity treatment, there is a critical need to investigate differences between MHO and metabolically unhealthy obesity, but informative studies are, thus far, very limited [7]. Obesity is associated with systemic and adipose tissue inflammation [12–14]. Excessive energy intake is thought to be a major contributor of obesity and leads to the accumulation of lipids in adipocytes and the expansion of adipose tissue. Hypertrophic adipocytes can produce pro-inflammatory cytokines, such as IL-6 and TNF, which leads to increased vascular permeability, circulating monocytes and the recruitment of cytotoxic T cells, initiating an inflammatory process that promotes insulin resistance [14, 15]. Recently, it has been reported that increased systemic and adipose tissue inflammation can differentiate obese women with impaired glucose tolerance from obese women with normal glucose tolerance [16]. Blüher and colleagues have shown that macrophage infiltration into visceral adipose tissue, higher inflammatory parameters and circulating adipokines can predict insulin resistance in morbidly obese patients [17]. However, only limited and partially discordant data are available on the association of systemic and adipose tissue inflammation with insulin resistance, especially in older patients.

In the present study, we focused on identifying relationships between the distribution of peripheral immune cell subsets, cytokine levels, and metabolic measures in a large non-clinical sample of older adults. Using data from BASE-II, we tested the hypothesis that individuals with obesity and insulin resistance have increased pro-inflammatory cytokines correlating with activated T cell subsets. The aim was to compare a cohort of obese participants with and without insulin resistance and a cohort of non-obese participants with and without insulin resistance.

## Results

### Characteristics of the sample

We analyzed 437 of 1671 older participants (60–84 years) of the BASE-II study, medically assessed at baseline, based on the availability of immunological parameters. The sample analyzed in this study was composed of participants with an equal gender distribution. Table 1 shows the median age, BMI, metabolic measurements (HbA1c, insulin sensitivity index (ISI_OGTT_), homeostasis model of assessment for insulin resistance (HOMA-IR)), glucose and insulin parameters measured in oral glucose tolerance tests (OGTT) and lipid parameters), hematologic parameters, and plasma C-reactive protein (CRP) levels of male and female participants. All hematologic parameters and CRP levels were within the normal range (NR). Obesity was noted in 28 of 181 men (15.5%) and 49 of 256 women (19.1%) (defined as BMI > 30 kg/m^2^), whereas 6.6% (women) and 11% (men) were diagnosed with type 2 diabetes. Participants with type 2 diabetes were ruled out for further analyses. However, 31.8% of the men and 28.8% of the women were considered IR at least to some extent, with insulin resistance being defined as ISI_OGTT_ < 4. Surprisingly, only 50% of all obese participants analyzed in this study had an ISI_OGTT_ < 4, and only 32% of all obese participants were considered truly IR, as assessed by HOMA-IR (HOMA-IR > 2.9).

**Table 1:** Characteristics of the BASE-II participants included in the present study

	Men (***n*** = 181)	Women (***n*** = 256)
Age [years]	70 (67–73)	68 (66–70)
BMI [NR 18–25 kg/m^2^]	26.9 (25.0–29.0)	25.8 (23.4–28.6)
Body weight [kg]	82.1 (76.4–90.5)	68.0 (62.0–76.3)
Height [cm]	175.7 (171.0–180.0)	163.2 (158.9–168)
HbA1c [NR < 5.7%]	5.5 (5.2–5.7)	5.4 (5.2–5.7)
HOMA-IR (NR < 1.9)	2.0 (1.4–3.0)	1.7 (1.2–2.5)
ISI_OGTT_ (NR > 4)^a^	5.6 (3.3–8.7)	5.7 (3.8–8.3)
Fasting PG (mg/dL)	102.5 (88.0–125.0)	104.0 (89.0–124.0)
PG in 75-g OGTT at 120 min (mg/dL)	94.0 (87.0–102.0)	89.0 (84.0–96.0)
Fasting Insulin in 75-g OGTT (mg/dL)	9.0 (6.3–12.3)	7.6 (5.7–10.4)
Insulin in 75-g OGTT at 120 min (mg/dL)	42.9 (25.6–72.4)	44.9 (29.7–67.3)
Triglycerides (NR </= 200 mg/dL)	104 (77–143)	91 (73–119)
HDL cholesterol (NR male/female =/> 35/45 mg/dL)	53 (45–65)	70 (57–79)
LDL cholesterol (NR < 130 mg/dL)	122 (100–147)	136 (112–162)
Cholesterol (NR < 200 mg/dL)	201 (177–228)	232 (202–256)
Obesity [n, %]	28 (15.5)	49 (19.1)
T2D [n, %]	20 (11.0)	17 (6.6)
HOMA-IR > 1.9 [n, %]	21 (12.1)	22 (9.1)
ISI_OGTT_ < 4 [n, %]^a^	51 (31.8)	65 (28.6)
CRP (NR < 5 mg/L)	1.2 (0.7–2.3)	1.2 (0.6–2.0)
Basophils (NR 0–0.2 G/L)	0.05 (0.05–0.05)	0.05 (0.05–0.05)
Eosinophils (NR 0.02–0.5 G/L)	0.18 (0.11–0.24)	0.15 (0.10–0.220)
Leukocytes (NR 3.9–10.5 G/L)	5.7 (4.7–6.7)	5.5 (4.6–6.4)
Monocytes (NR 0.1–0.9 G/L)	0.45 (0.36–0.53)	0.35 (0.29–0.43)
Neutrophils (NR 1.5–7.7 G/L)	3.2 (2.7–4.0)	3.1 (2.5–3.7)

Categorical data are presented as percentages. None of the continuous variables were normally distributed; thus, values are presented as median and interquartile range. *BMI* body mass index, *HOMA-IR* homeostasis model assessment of insulin resistance. *ISI*_*OGTT*_ insulin sensitivity index, *NR* normal range, *OGTT* oral glucose tolerance test, *PG* plasma glucose, *T2D* type 2 diabetes. ^a^ISI_OGTT_ was not assessed for participants with type 2 diabetes

### Impaired insulin sensitivity is associated with increased T cell senescence

**Fig. 1:**
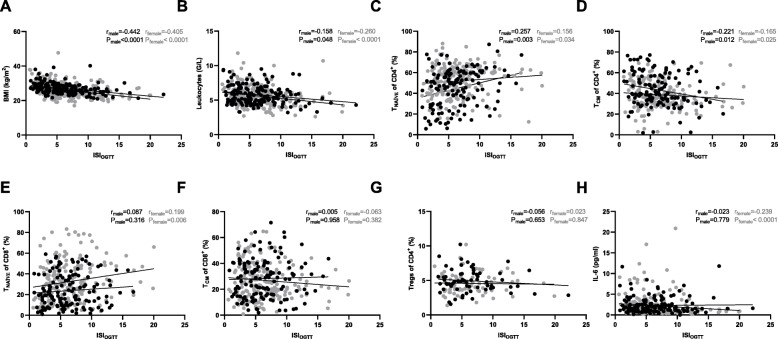
Associations between ISI_OGTT_, immune cell frequencies, and cytokine levels in older participants. **a**-**h** Insulin sensitivity index (ISI_OGTT_) was correlated with body mass index (BMI), leukocytes, naïve CD4^+^ T cells (T_NAIVE,_ CD45RA^+^CCCR7^+^CD27^+^CD28^+^), central memory CD4^+^ T cells (T_CM,_ CD45RA^−^ CD27^+^CD28^+^), naïve CD8^+^ T cells (T_NAIVE,_ CD45RA^+^CCR7^+^ CD27^+^CD28^+^), central memory CD4^+^ T cells (T_CM,_ CD45RA^−^CD27^+^CD28^+^), regulatory CD4^+^ T (Treg) cells (FoxP3^+^CD25^high^), and IL-6 levels. Spearman correlation analysis was conducted. * *p* < 0.05. Each dot represents one participant. Male participant (black). Female participant (grey)

As expected, the BMI was negatively associated with ISI_OGTT_ in both male and female participants (Fig. 1a). The ISI_OGTT_ also correlated negatively with the total number of leukocytes, obtained from complete blood count results (Fig. 1b), in line with previous results from another study of ours [18]. To assess the association of metabolic parameters (BMI, HOMA-IR, ISI_OGTT_) with systemic leukocyte subpopulations, we analyzed multiparameter flow cytometric data derived from blood samples of older participants. To analyze gender-specific differences, we here calculated each correlation independently for men and women. Whereas the percentages of major mononuclear leukocyte subsets (CD4^+^ and CD8^+^ T cells, CD19^+^ B cells, CD56^+^NK cells, and CD14^+^monocytes) did not significantly correlate with BMI, HOMA-IR or ISI_OGTT_, we found that the percentages of naïve (CD45RA^+^CCR7^+^CD27^+^CD28^+^) CD4^+^ and CD8^+^ T cell subsets (the latter only within the female subgroup) were positively associated with ISI_OGTT_ and negatively associated with HOMA-IR in older participants, whereas the percentage of central memory (CD45RA^−^CD27^+^CD28^+^) CD4^+^ T cells was negatively associated with ISI_OGTT_ and positively associated with HOMA-IR (Fig. 1c-e, Supplementary Table 1). However, neither men nor women exhibited a significant correlation between the percentage of central memory CD8^+^ T cells and the ISI_OGTT_ (Fig. 1f). The percentage of effector memory (CD45RA^−^CD27^−^CD28^−^) CD8^+^ T cells correlated positively with HOMA-IR, but other T cell subsets (exhausted PD1^+^ T cells or terminally differentiated effector memory T cells) did not show significant associations with metabolic measures (Supplementary Table 1). While regulatory T (Treg) cells have been reported to play a role in regulating obesity-induced adipose tissue inflammation [19], the percentage of circulating FoxP3^+^CD25^high^ Treg cells within the CD4^+^ T cell subset was not associated with BMI, HOMA-IR or ISI_OGTT_ in this study (Fig. 1g, Supplementary Table 1). However, CD8^+^ FoxP3^+^ Treg cells, which have been reported recently to be critical in prevention of autoimmune-mediated diabetes, correlated positively with BMI (data not shown). The different subsets of B cells did not correlate significantly with metabolic measures. Whereas the percentage of CD14^−^CD16^+^ monocytes correlated inversely with HOMA-IR and ISI_OGTT_, other monocyte subsets did not exhibit significant correlations (Supplementary Table 1). Additionally, we correlated cytokine levels (IL-6, IL-10, TNF, IL-1ß) with metabolic measures. As described previously [20], the seminal inflammatory marker IL-6 was negatively associated with ISI_OGTT_ in female participants (Fig. 1h), but the associations between IL-10, TNF, IL-1ß and metabolic measures were not significant (data not shown). CD57 has been proposed as a marker for T cell senescence, as its expression is associated with impaired proliferative capacity and other characteristics of senescence [21]. However, CD57 can also define activated immunoregulatory-like cells [22]. Here, we found a positive association of CD57 expression on central memory CD8^+^ T cells with BMI, whereas CD57^+^ effector memory CD8^+^ T cells correlated positively with HOMA-IR (Supplementary Table 1). With age, the distribution of circulating T cells at different stages of differentiation changes drastically, as a result of the minimal production of naïve T cells, and the accumulation of senescent-like CD28^−^CD57^+^CD8^+^T cells, which have been shown to be associated with reduced immune response to pathogens in the elderly [22]. This could contribute to increased severity of infections in the older population [23]. Taken together, our findings thus imply that insulin resistance is associated with a higher “age” of circulating T cells. Since the serostatus of CMV can affect the phenotype of immune cell subsets as well [24], we additionally correlated the BMI and ISI_OGTT_ with immune cell parameters from CMV seronegative and seropositive participants. We found that the correlation coefficients were influenced by the CMV serostatus and that some significant correlations between T cell subsets, BMI and ISI_OGTT_ were found in CMV^+^, but not CMV^−^ participants.

### T cells have a more activated phenotype in IS versus IR obese individuals

**Fig. 2:**
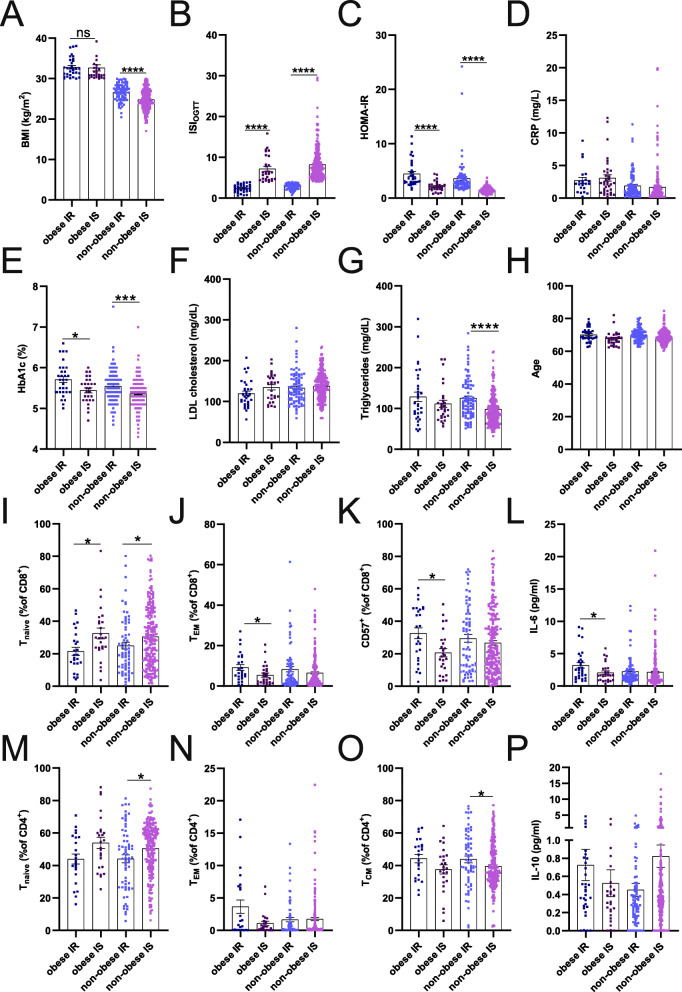
Comparison of the distributions of metabolic parameters, immune cell subpopulation proportions, and cytokine levels in obese and non-obese insulin resistant (IR) and insulin sensitive (IS) participants. **a**-**p** Mann-Whitney U test was conducted to estimate differences between obese (BMI > 30 kg/m^2^) IS and IR, and non-obese (BMI < 30 kg/m^2^) IS and IR participants. **a**-**h** BMI (**a**), ISI_OGTT_ (**b**), HOMA-IR (**c**), CRP levels (**d**), HbA1c (**e**), LDL cholesterol (**f**), triglycerides (**g**), and age (**h**) were compared between groups. **i**-**p** (**i**) Naïve CD8^+^ (T_NAIVE,_ CD45RA^+^CCR7^+^ CD27^+^CD28^+^), (**j**) effector memory CD8^+^ T cells (T_EM,_ CD45RA^−^ CD27^−^CD28^−^), (**k**) CD57+ CD8+ T cells, (**l**) IL-6 levels, (**m**) naïve CD4^+^ T cells (T_NAIVE,_ CD45RA^+^CCR7^+^ CD27^+^CD28^+^), (**n**) effector memory CD4^+^ T cells (T_EM,_ CD45RA^−^ CD27^−^CD28^−^), (**o**) central memory CD4^+^ T cells (T_CM,_ CD45RA^−^ CD27^+^CD28^+^), (**p**) and IL-10 levels were compared between groups. Data are shown as mean ± SEM. * p < 0.05. **** *p* < 0.0001

To better characterize the immunological differences between IS, who are considered metabolically healthy, and IR (considered metabolically unhealthy) obese and non-obese participants of this study, we stratified the cohort into four groups by the ISI_OGTT_. The BMI was significantly lower in non-obese IS participants (Fig. 2a) but did not differ significantly between IS and IR obese participants. As expected, ISI_OGTT_, HOMA-IR and HbA1c were significantly different between IS and IR obese and non-obese participants (Fig. 2b, c and e), whereas CRP, triglyceride and LDL cholesterol levels, and age did not differ significantly between groups (Fig. 2d and f-h). The frequencies of naïve CD8^+^ T cells were significantly higher in obese and non-obese IS compared to IR participants, whereas the frequencies of effector memory and CD57^+^ antigen-experienced and differentiated CD8^+^ T cells were significantly lower in obese IS compared to IR individuals (Fig. 2i-k). Additionally, IL-6 levels, which have previously been reported to promote insulin resistance [20], were higher in obese IR than IS individuals (Fig. 2l). In the CD4^+^ T cell compartment, frequencies of naïve CD4^+^ T cells were higher and frequencies of central memory CD4^+^ T cells were lower in IS versus IR non-obese individuals, while we did not see any significant differences in the obese group (Fig. 2m-o, Supplementary Table 3). No significant differences between groups were found for other T cell subsets, B cells, NK cells, monocytes or CD4^+^ Treg cells (Supplementary Table 3). In addition, the levels of the anti-inflammatory cytokine IL-10 (Fig. 2p), TNF and IL-1ß were not significantly different in the obese and non-obese subgroups (Supplementary Table 4). TNF and IL-1ß were undetectable in some participants, and these were excluded in further studies. These findings suggest that senescence of circulating T cells reflects a major difference between IS and IR participants in both obese and non-obese subgroups.

### The percentage of naïve CD4^+^ and CD8^+^ T cells predicts impaired insulin sensitivity

Next, we tested the association of ISI_OGTT_ with systemic leukocyte subsets and cytokine levels in multivariable linear regression models. The frequencies of T cells, Treg cells, monocytes, B cells, and NK cells were not significantly associated with ISI_OGTT_, neither after adjustment for sex and BMI (model 1) nor with additional adjustment for the morbidity index and cytomegalovirus (CMV) status (model 2). More details of these results that are in line with our findings that the proportions of major innate and adaptive leukocyte subsets (T cells, Treg cells, monocytes, B cells, and NK cells) were not significantly different in obese or non-obese IR and IS participants can be found in Supplementary Table 5. T stem cell-like memory T cells (TSCM, defined as CD45RA^+^CCR7^+^CD27^+^CD28^+^CD95^+^) were also not significantly associated with ISI_OGTT_ (Supplementary Table 5). Table 2 summarizes the regression analyses of naïve, central, effector, and terminally differentiated effector memory CD4^+^ and CD8^+^ T cells on ISI_OGTT_. Whereas central and terminally differentiated effector memory CD4^+^ and CD8^+^ T cells did not predict ISI_OGTT_, naïve CD4^+^ and CD8^+^ T cells, and central memory CD4^+^ T cells were significantly associated with ISI_OGTT_. Of note, this significant association was independent of sex, BMI, and even after adjustment for the morbidity index and CMV status in model 2, the associations of naïve CD4^+^ T cells and ISI_OGTT_ remained significant (Table 2, Supplementary Table 5). We replicated these findings using an additional binary logistic regression model (Supplementary Table 6). To account for potential selectivity based on high age, we also added a sensitivity analysis, and ruled out participants, that were aged 80 years or older (8 participants, age 80–84 years). None of those participants were obese and the results remained unchanged (Supplementary Table 7). CMV was included in this analysis because latent infection with this herpesvirus strongly influences circulating naïve and memory T cell phenotypes [25]. Circulating IL-6 and IL-10 levels were not significantly associated with ISI_OGTT_ (data not shown). Taken together, these data indicate that the differentiation and activation state of CD4^+^ and CD8^+^ T cells is strongly associated with insulin resistance.

**Table 2:**
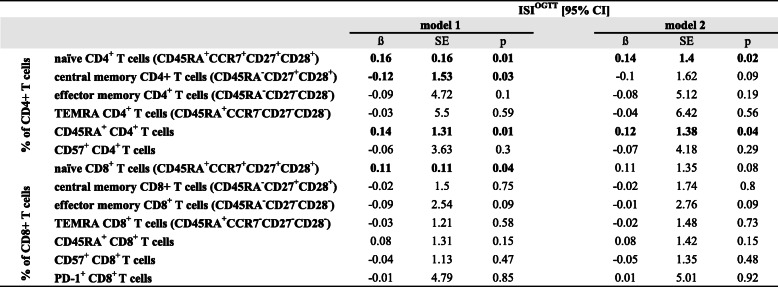
Association of immune cell subsets with ISI_OGTT_

Linear regression adjusted for sex, BMI (model 1), and linear regression adjusted for sex, body mass index (BMI), morbidity index and CMV status (model 2). SE (standard error). Significant associations are highlighted in bold

## Discussion

The prevalence of obesity has dramatically increased in all age groups in recent years, but obesity rates among older adults are even higher. Increasing age has been shown to be associated with lower prevalence of MHO [10]. Recently, it has been reported that obesity-related comorbidities and conditions mirror those of aging and age-related diseases [26]. Obesity and aging can lead to chronic low-grade inflammation and an increased incidence of chronic inflammatory diseases due to dysregulated immune responses [27–31]. Macrophages seem to be primarily involved in obesity-associated inflammation, changing their phenotype from “alternatively activated” to “inflammatory” macrophages [32, 33]. Moreover, other components of the innate immune system, mast cells, neutrophils, and dendritic cells, have been shown to exacerbate insulin resistance [34–36], whereas eosinophils and type 2 innate lymphoid cells can protect against adipose tissue and islet inflammation [35]. More recent work focused on adaptive immune responses in obesity-induced systemic and adipose tissue inflammation. T cells, including CD8^+^ T cells, Th1, Th17, as well as B cells, can exacerbate inflammation, whereas Treg cells and Th2 cells can dampen inflammation and protect against insulin resistance [37–39]. Several human studies investigated the association of inflammatory parameters with impaired insulin sensitivity. In particular, it has been found that the helper T cell composition in peripheral blood correlates significantly with the HOMA-IR [40] and other measures of adiposity, inflammation and glucose intolerance, whereas circulating Treg cells were reduced in obese subjects, and might identify individuals at increased risk for cardiovascular comorbidities [41, 42]. Moreover, a distinct phenotype of Treg cells has been characterized in human obese omental adipose tissue [43]. Another recent study revealed an impaired NK cell phenotype and NK cell subset alterations in obese individuals [44]. Reduced circulating Treg cell numbers were detected in obese compared with non-obese study participants [41], and another study identified a significant inverse correlation of Th2 cells in peripheral blood with systemic insulin resistance [12]. Additionally, our group found recently that insulin resistance correlates significantly with a shift in the ratio of naïve and differentiated memory CD4^+^ and CD8^+^ T cells in abdominal subcutaneous adipose tissue in female obese subjects [18]. Furthermore, it has been found that peripheral frequencies of T-helper (Th)22 cells and IL-22 levels were increased in obese subjects with or without type 2 diabetes compared with lean subjects, and that Th22 cell frequencies correlated positively with HOMA-IR [40]. These findings were confirmed in another study, showing that Th22 and Th17 cells were elevated in abdominal subcutaneous adipose tissue from metabolically abnormal IR obese compared with metabolically normal IS obese subjects [45]. Although the association of obesity with systemic low-grade inflammation is well established, immunological studies on the characteristics of IS versus IR metabolically unhealthy obese individuals are limited.

Here, we have investigated the association of insulin resistance and immunological parameters in a sample of 437 older participants of the BASE-II study. We analyzed peripheral blood immune cell subsets and cytokine levels with multiparameter flow cytometry analysis. We found that frequencies of naïve CD4^+^ and CD8^+^ T cells correlated positively with ISI_OGTT_, but negatively with BMI and HOMA-IR, whereas frequencies of central memory CD4^+^ T cells correlated negatively with ISI_OGTT_, but positively with BMI and HOMA-IR. Additionally, the percentage of highly differentiated effector memory CD8^+^ T cells was positively associated with HOMA-IR, and the expression of CD57, a surface marker putatively associated with impaired proliferation capacity, immunoregulation, and cell senescence [21], correlated positively with BMI and HOMA-IR in older participants of this study. However, the expression of PD-1, a characteristic marker for T cell exhaustion, on CD8^+^ T cells was not associated with metabolic measures (Supplementary Table 1). In line with previous reports [20, 46], we also identified a positive association of systemic IL-6 levels with insulin resistance, whereas other cytokines (IL-1ß, TNF, IL-10) did not correlate significantly (Fig. 1). Cytokines can be used as target biomarkers for immune response and inflammaging. Recently, IL-6, which is known to be a main stimulator of the production of acute-phase proteins, was shown to be elevated with advanced age in a sample of healthy people and correlated with BMI and CRP levels [47]. IL-6 has pleiotropic effects on inflammation, immune response, and hematopoiesis, but dysregulated synthesis of IL-6 plays a pathological effect on chronic inflammation and autoimmunity. To address the association of MHO with insulin resistance, we divided the study participants in obese and non-obese IR and IS subgroups, based on ISI_OGTT_. The increased frequencies of peripheral blood CD4^+^ T cells in IS obese and non-obese individuals were accompanied by a selective increase of naïve CD4^+^ T cells. Similarly, in the CD8^+^ T cell compartment, the frequencies of naïve T cells were higher in IS obese and non-obese subgroups, whereas effector memory T cells were significantly lower. Altogether, the CD4^+^ and CD8^+^ T cell compartment was skewed towards a senescent-like T cell phenotype in IR subjects (Fig. 2, Supplementary Table 3–4). Additionally, the frequencies of naïve CD4^+^ and CD8^+^ T cells were predictive for ISI_OGTT_, and the relationship of ISI_OGTT_ with naïve CD4^+^ T cells remained significant after adjustment for sex, BMI, clinical conditions (morbidity index) and CMV-serostatus (Table 2).

Here, we investigated the association of insulin resistance with immune cell parameters in older study participants and defined IS participants that were not treated with antidiabetic medication as “metabolically healthier”, but the definition of MHO is still controversial. Recently, the following criteria have been proposed in addition to the diagnosis of obesity (BMI > 30 kg/m^2^) fasted serum triglycerides ≤1.7 mmol/l (≤150 mg/dl); HDL cholesterol serum concentrations > 1.0 (> 40 mg/dl) (in men) or > 1.3 mmol/l (> 50 mg/dl) (in women); systolic blood pressure (SBP) ≤130 mmHg; diastolic blood pressure ≤ 85 mmHg; fasting blood glucose ≤6.1 mmol/l (≤100 mg/dl); no drug treatment for dyslipidemia, diabetes, or hypertension; and no cardiovascular disease manifestation [7]. The number of BASE-II participants that would have met all criteria was extremely low, which can be attributed to their age, for which reason we only assessed parameters for insulin sensitivity and insulin resistance for further analyses. Interestingly, frequencies of systemic naïve CD4^+^ and CD8^+^ T cells of older participants of the BASE-II study, were positively associated with HDL cholesterol serum concentrations, whereas associations with fasting blood glucose and fasted serum triglycerides were negative (data not shown).

Our study has several important limitations. First, we could only include a small subgroup of BASE-II study participants based on the availability of flow cytometric data and cytokine level measurements of blood samples. Although the distribution of the analyzed subgroup is similar to the whole cohort (e.g. equal gender distribution), the sample size is rather small after further subdivision into obese and non-obese, IS and IR groups, and the different numbers of IS (*n* = 243) versus IR (*n* = 84) non-obese participants could limit the power to detect significant associations. However, the sample size of obese IS (*n* = 28) and IR (*n* = 32) participants is similar, and regarding the multiparameter flow cytometry analysis we conducted, the sample size is still reasonable. Moreover, the relative homogeneity of the participants with regard to age (65–80 years) could strengthen our study; age-related changes in immune function and T cell alterations have been described previously [25, 28].

Second, we measured insulin sensitivity using HOMA-IR and ISI_OGTT_ obtained from OGTT, whereas the hyperinsulinemic-euglycemic clamp technique is considered the most reliable method available for estimating insulin resistance and is used as reference standard. In the present study, only half of the obese subgroup was considered IR with ISI_OGTT_ < 4 and 32% of the obese subgroup was considered IR with HOMA-IR < 2.9. On the other hand, 19% of the non-obese subgroup was considered IR with ISI_OGTT_ < 4, and 4.6% with HOMA-IR > 2.9, which might be explained by significantly higher BMI in the IR non-obese subgroup. Due to significant inter laboratory variations in insulin assays, the normal range of these parameters needs to be established for each laboratory and could also explain differences in the percentages of participants considered IR, here. However, the measurements of HOMA-IR and ISI_OGTT_ are minimally invasive, and, thus, still suitable for clinical uses. Moreover, these surrogate parameters of insulin resistance are widely used in observational studies which allows for comparison between different studies.

Third, we assessed peripheral blood immune cell frequencies in this study, which often correlate with immune cell profiles in adipose tissues [12], but further investigation on immune cell parameters in abdominal subcutaneous and visceral adipose tissue and adipose tissue dysfunction [48] is needed to elucidate biological mechanisms linking obesity to insulin resistance.

## Conclusions

In conclusion, our study underscores the role of immunological parameters in the differentiation of IS from IR individuals with obesity. Our results suggest that the peripheral blood T cell compartment of IS individuals is characterized by higher frequencies of naïve CD4^+^ and CD8^+^ T cells, whereas differentiated and activated memory CD4^+^ and CD8^+^ T cell frequencies are lower than in IR individuals. Further studies are needed to explore mechanisms underlying the relationship between T cell senescence and insulin resistance in obesity, and to characterize immunological parameters of MHO to guide risk-stratified obesity treatment.

## Methods

### Study population

We analyzed a subgroup of 437 participants of the BASE-II study selected based on the availability of immune cell parameters, randomly selected from all 1600 participants (Table 1). BASE-II is a prospective multidisciplinary and multi-institutional study that investigates factors associated with aging trajectories in Berlin [49–51]. Phenotypic assessments include factors related to geriatrics and internal medicine, immunology, genetics, psychology, sociology, and economics. Initial medical assessments included 2171 participants (∼75% aged 60–84 years and ∼25% aged 20–35 years) [52]. Based on the BMI and the ISI_OGTT_, we divided the participants into four groups: obese and non-obese (BMI > 30 kg/m^2^ and BMI < 30 kg/m^2^) and IR (ISI_OGTT_ < 4) and IS (ISI_OGTT_ > 4). Medication use was initially assessed by a study physician. Patients with antidiabetic medications like metformine, sulfonylureas, thiazolidinediones and metiglinides were ruled out from the analyses. All participants gave written informed consent to the study protocol which was approved by the Ethics Committee of the Charité-Universitätsmedizin Berlin (number of the ethical approval: EA2/ 029/09).

### Biochemical measurements

A peripheral venous blood sample of all BASE-II participants was drawn in the morning after an overnight fast (> 8 h) and kept at 4 °C until analysis on the same day. Serum concentrations of total cholesterol, low-density lipoprotein, cholesterol, high-density lipoprotein cholesterol, and triglycerides were measured using enzymatic colorimetric tests or photometric measurements. Additionally, venous blood samples were taken for complete blood count. Glucose levels (fasting and 2-h post load) were measured using photometric methods and insulin levels were determined by an electrochemiluminescence immunoassay (Elecsys® Insulin, Cobas/Roche). HbA1c was measured using high-performance chromatography (VARIANT II TURBO HbA1c Kit – 2.0, Bio-Rad). Insulin resistance was determined using the HOMA-IR (calculated as the product of fasting glucose and fasting insulin divided by 22.5). Moreover, insulin sensitivity was estimated by calculating ISI_OGTT_ based on the work of Matsuda and colleagues [53]. CRP levels were determined using an immunological turbidity assay (cobas/Roche, Rotkreuz, Switzerland), as described previously [54].

### Additional data

Body weight was measured with a portable electronic scale to the nearest 0.1 kg and height was determined to the nearest 0.1 cm by using an electronic weighing and measuring station (seca 764, seca, Hamburg, Germany). Weight and height were used for calculating the BMI, kg/m^2^. Morbidity was assessed as a morbidity index based on most of the categories of the comorbidity index originally described by Charlson and collaborators [55], which is a weighted sum of moderate to severe, mostly chronic illnesses, including cancer (e.g., lymphoma) and cardiovascular (e.g., congestive heart failure) and metabolic diseases (e.g., diabetes mellitus). The morbidity index used in BASE-II has been described previously in detail [56].

### Flow cytometry on peripheral blood mononuclear cells

Venous blood was taken from the participants of the BASE-II study during medical examinations at the Charité in Berlin and sent to the BASE-II partner site at the University Tübingen in EDTA tubes (7 ml) packed in iso-containers, to minimize temperature variations. The peripheral blood mononuclear cells (PBMC) were further isolated under sterile conditions and frozen at − 196 °C in liquid nitrogen until further processing. Flow cytometry surface staining was performed as described previously [57]. First, PBMCs were treated with human Ig, GAMUNEX (Bayer, Leverkusen, Germany), and ethidium monoazide (EMA) bromide (MoBiTec GmbH, Göttingen, Germany) for 10 min on ice to block nonspecific binding of antibodies and label dead cells. Cells were stained for 20 min on ice. Monoclonal antibodies used for flow cytometry are listed in Supplementary Table 8. Cells were then washed and analyzed immediately on a LSR II cytometer (BD, Heidelberg) with FACSDiva software (BD Biosciences). Data were analyzed using FlowJo 7.6.5 software (Tree Star, Portland, USA). T-cell subsets were characterized according to previously published models [58]. In brief, viable lymphocytes were gated within the CD3^+^gate and then selected for either CD8^+^ or CD4^+^ T cell subsets, which were further subdivided into main T cell phenotypes (T_Naive_, T_CM_, T_EM_ and T_EMRA_) characterized by expression of CD45RA, CCR7, CD27 and CD28. The distinct subsets of CD4^+^ and CD8^+^ T cells were defined as follows: naïve T cells (T_NAIVE,_ CD45RA^+^CCR7^+^CD27^+^CD28^+^), central memory T cells (T_CM,_ CD45RA^−^CD27^+^CD28^+^), effector memory T cells (T_EM,_ CD45RA^−^ CD27^−^CD28^−^), and terminally differentiated effector memory T cells (T_EMRA,_ CD45RA^+^CCR7^−^CD27^−^CD28^−^). Additionally, T_Naive_ cells have been gated for CD95 expression to identify TSCM (CD45RA^+^CCR7^+^CD95^+^CD27^+^CD28^+^) cells and PD-1 (CD279) expression was determined within the CD8^+^ population. Treg cells were defined as FoxP3^+^CD25^high^ cells within the CD4^+^ or CD8^+^ T cell subset, as described previously [59]. The gating strategy for T cells is shown in Supplementary Figure 1 and Supplementary Figure 2. B cells were defined as viable CD45^+^CD19^+^CD3^−^ cells and were further divided into naïve and memory B cells based on the expression of IgD and CD27. NK cells were defined as viable CD45^+^CD14^−^CD3^−^CD56^+^ cells, and monocytes were defined as viable CD45^+^CD14^+^ cells. The gating strategy for B cells has been reported elsewhere [57]. Flow cytometry staining and data analysis were performed on blinded samples.

### Cytokine analysis

Serum cytokine levels were determined as described previously [31, 60]. Briefly, cytokine levels of IL-1ß, IL-6, IL-10 and TNF were determined using the high sensitivity CBA flex system (BD Biosciences), according to the manufacturer’s instructions. Samples were measured (BD LSR-II) and consistent performance was assured using BD CS&T beads.

### CMV serology

Anti-CMV IgG titres were analyzed using a CMV IgG Kit (Omega Diagnostics Group, Scotland, UK) as described previously [54]. IgG levels were measured using a semi-quantitative approach, according to the manufacturer’s instructions.

### Statistical analysis

The results are shown as median 25th, and 75th percentile for continuous variables or as absolute numbers and percentages for categorical variables unless otherwise noted. All statistical analyses were performed with IBM SPSS Statistics software package, version 25 and GraphPad Prism version 8 (GraphPad Software, San Diego, CA). A one-sample Kolmogorov-Smirnov test was used to test variables for Gaussian distribution. The spearman rank correlation coefficient was used for analyzing bivariate correlations between the ISI_OGTT_ and immune cell subsets and cytokine levels. The Mann-Whitney *U* test was applied to estimate differences between groups. Associations of systemic immune cell subsets and cytokine levels with metabolic measures were analyzed by linear regression models adjusted for sex, BMI, CMV-serostatus and the morbidity index. Because data analyses were exploratory no adjustment was made for multiple testing and *p* values were interpreted descriptively. An acceptable level of statistical significance was established a priori at *p* < 0.05.

## Supplementary Information

**Additional file 1: Supplementary Table 1–8**. The association of adaptive immune cell phenotypes with metabolically healthy obesity.

**Additional file 2: Supplementary Figure 1.** Gating strategy for CD4^+^ T cells. **Supplementary Figure 2** Gating strategy for CD8^+^ T cells.

## Data Availability

The datasets used and/or analyzed during the current study are available from the corresponding author on reasonable request.

## Abbreviations

BASE-II: Berlin Aging Study II
BMI: Body mass index
HOMA-IR: Homeostasis model of assessment for insulin resistance
IR: Insulin resistant
IS: Insulin sensitive
ISI_OGTT_: Insulin sensitivity index
MHO: Metabolically healthy obesity
NR: Normal range
OGTT: oral glucose tolerance test
CRP: Plasma C-reactive protein
Treg cells: Regulatory T cells
TSCM: T Stem cell-like memory T cells
