# Supplementary material for: T cell phenotypes associated with insulin resistance: results from the Berlin Aging Study II
Source: Immun Ageing. 2020 Dec 21;17:40. doi: 10.1186/s12979-020-00211-y (PMC7751110; doi:10.1186/s12979-020-00211-y)
Supplement: Supplementary file 1 — Additional file 1: Supplementary Table 1–8. The association of adaptive immune cell phenotypes with metabolically healthy obesity. [file 12979_2020_211_MOESM1_ESM.docx]

**T cell phenotypes associated with insulin resistance: Results from the Berlin Aging Study II**

**Julia Sbierski-Kind^1^, David Goldeck^2^, Nikolaus Buchmann^3^, Joachim Spranger^1,4,5^, Hans-Dieter Volk^6^, Elisabeth Steinhagen-Thiessen^1^, Graham Pawelec^7,8^, Ilja Demuth^1,6^, Dominik Spira^1^**

^1^Charité-Universitätsmedizin Berlin, Corporate Member of Freie Universität Berlin, Humboldt-Universität zu Berlin, and Department of Endocrinology and Metabolism, Berlin Institute of Health, Berlin, Germany.

^2^Fairfax Centre, Kidlington, United Kingdom.

^3^Clinic for Cardiology, Charité Universitätsmedizin Berlin, Berlin, Germany.

^4^Center for Cardiovascular Research (CCR), Department of Endocrinology and Metabolism, Charité-Universitätsmedizin Berlin, Berlin, Germany.

^5^German Center for Cardiovascular Research (DZHK), partner site Berlin, Berlin, Germany.

^6^Berlin Institute of Health (BIH) Center for Regenerative Therapies (BCRT), Charité - Universitätsmedizin Berlin, Berlin, Germany; Berlin Center for Advanced Therapies (BeCAT), Charité - Universitätsmedizin Berlin, Berlin, Germany; Institute of Medical Immunology, Charité - Universitätsmedizin Berlin, Berlin, Germany.

^7^Department of Immunology, University of Tübingen, Tübingen, Germany.

^8^Health Sciences North Research Institute, Sudbury, ON, Canada.

Address correspondence to Julia Sbierski-Kind, MD, Department of Endocrinology and Metabolism, Charité-Universitätsmedizin Berlin, Berlin, Germany. Chariteplatz 1, 10117 Berlin, Deutschland.

**Present address**

University of California, San Francisco
Dept. of Laboratory Medicine
HSW1201U, Box 0451
513 Parnassus Ave
San Francisco, CA 94143-0451

Julia.Sbierski-Kind@ucsf.edu

**Supplementary Table 1**

**Association between BMI, HOMA-IR, ISI_OGTT_ and immune cell frequencies in elderly participants.**

Spearman correlation analysis was conducted. * p < 0.05. ** p < 0.01. Body mass index (BMI). Homeostatic model assessment of insulin resistance (HOMA-IR) Insulin sensitivity index (ISI_OGTT_).

**Supplementary Table 2**

Spearman correlation analysis was conducted for CMV^-^ and CMV^+^ participants. * p < 0.05. ** p < 0.01. Body mass index (BMI). Homeostatic model assessment of insulin resistance (HOMA-IR) Insulin sensitivity index (ISI_OGTT_).

**Supplementary Table 3**

**Comparison of the distributions of immune cell subpopulation proportions in obese and non-obese insulin resistant and non-insulin resistant participants.**

Mann-Whitney U test was conducted to estimate differences between obese (BMI > 30 kg/m^2^) non-insulin resistant and insulin resistant, and non-obese (BMI < 30 kg/m^2^) non-insulin resistant and insulin resistant participants. Data are given as median and interquartile range. Body mass index (BMI). Insulin sensitivity index (ISI_OGTT_).

**Supplementary Table 4**

**Comparison of cytokine levels in obese and non-obese insulin resistant and non-insulin resistant participants.**

Mann-Whitney U test was conducted to estimate differences between obese (BMI>30 kg/m^2^) non-insulin resistant and insulin resistant, and non-obese (BMI < 30 kg/m^2^) non-insulin resistant and insulin resistant participants. Data are given as median and interquartile range. Body mass index (BMI). Insulin sensitivity index (ISI_OGTT_).

**Supplementary Table 5**

**Association of immune cell subsets with ISI_OGTT._**

Linear regression adjusted for sex, BMI (model 1), and linear regression adjusted for sex, BMI, morbidity index and CMV status (model 2). SE (standard error). Significant associations are highlighted in bold.

**Supplementary Table 6**


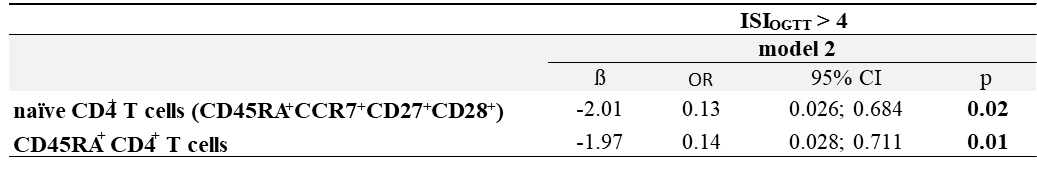
**Logistic regression analysis displaying the association of immune cell subsets with ISI_OGTT._**

Logistic regression adjusted for sex, BMI, morbidity index and CMV status (model 2). OR (odds ratio). CI (confidence interval). Significant associations are highlighted in bold.

**Supplementary Table 7**

**Linear regression analysis displaying the association of immune cell subsets with ISI_OGTT._**

Linear regression adjusted for sex, BMI, morbidity index and CMV status (model 2) and analyzed after ruling out subjects aged 80 years or older (8 subjects, age 80-84 years). CI (confidence interval). Significant associations are highlighted in bold.

**Supplementary Table 8**

**Antibody Table**

**REAGENT or RESOURCE**

**SOURCE**

**IDENTIFIER**

Anti-human CD45RA BUV 421 (clone HI100)

BioLegend

Cat#304130; AB_10965547

Anti-human CD3 Pacific Orange

Life Technologies

Cat#CD0330; AB_2536469

Anti-human CD25 PE (clone M-A251)

BD Biosciences

Cat#555432; AB_395826

Anti-human CD4 PercP (clone SK3)

BD Biosciences

Cat#345770;

Anti-human FoxP3 AF 647 (clone 259D/C7)

BD Biosciences

Cat#560045; AB_1645411

Anti-Human CD8 APC-H7 (clone SK1)

BD Biosciences

Cat#560179; AB_1645481

Anti-human CCR7(clone CD197)

BD Biosciences

Cat#557648

Anti-human CD45 V500 (clone HI30)

BD Horizon

Cat#560777; AB_1937324

Anti-human CD3 APC (clone UCHT1)

BioLegend

Cat#300412; AB_314066

Anti-Human CD14 APC-H7 (clone M5E2)

BD Biosciences

Cat#561384;

Anti-Human CD56 BUV 605 (clone NCAM16.2)

BD Horizon

Cat#562780; AB_2728700

Anti-Human CD16 BUV 711 (clone 3G8)

BioLegend

Cat#302043; AB_11219184

Anti-Human CD279 (clone EH12.2H7)

BioLegend

Cat#329937

Anti-Human CD95 PE-Cy7 (clone DX2)

BD Biosciences

Cat#561636

Anti-Human CD57 FITC (clone HNK-1)

BD Biosciences

Cat#347393

Anti-Human CD19 V500 (clone HIB19)

BD Biosciences

Cat#561121

Anti-Human CD27 APC (clone O323)

BioLegend

Cat#302809

Anti-Human CD28 PE (clone CD28.2)

BD Biosciences

Cat#561793

Anti-Human IgD PE (clone IA6-2)

BioLegend

Cat#348203; AB_10550096
