# Supplementary figures and images for: T cell phenotypes associated with insulin resistance: results from the Berlin Aging Study II
Source: Immun Ageing. 2020 Dec 21;17:40. doi: 10.1186/s12979-020-00211-y (PMC7751110; doi:10.1186/s12979-020-00211-y)

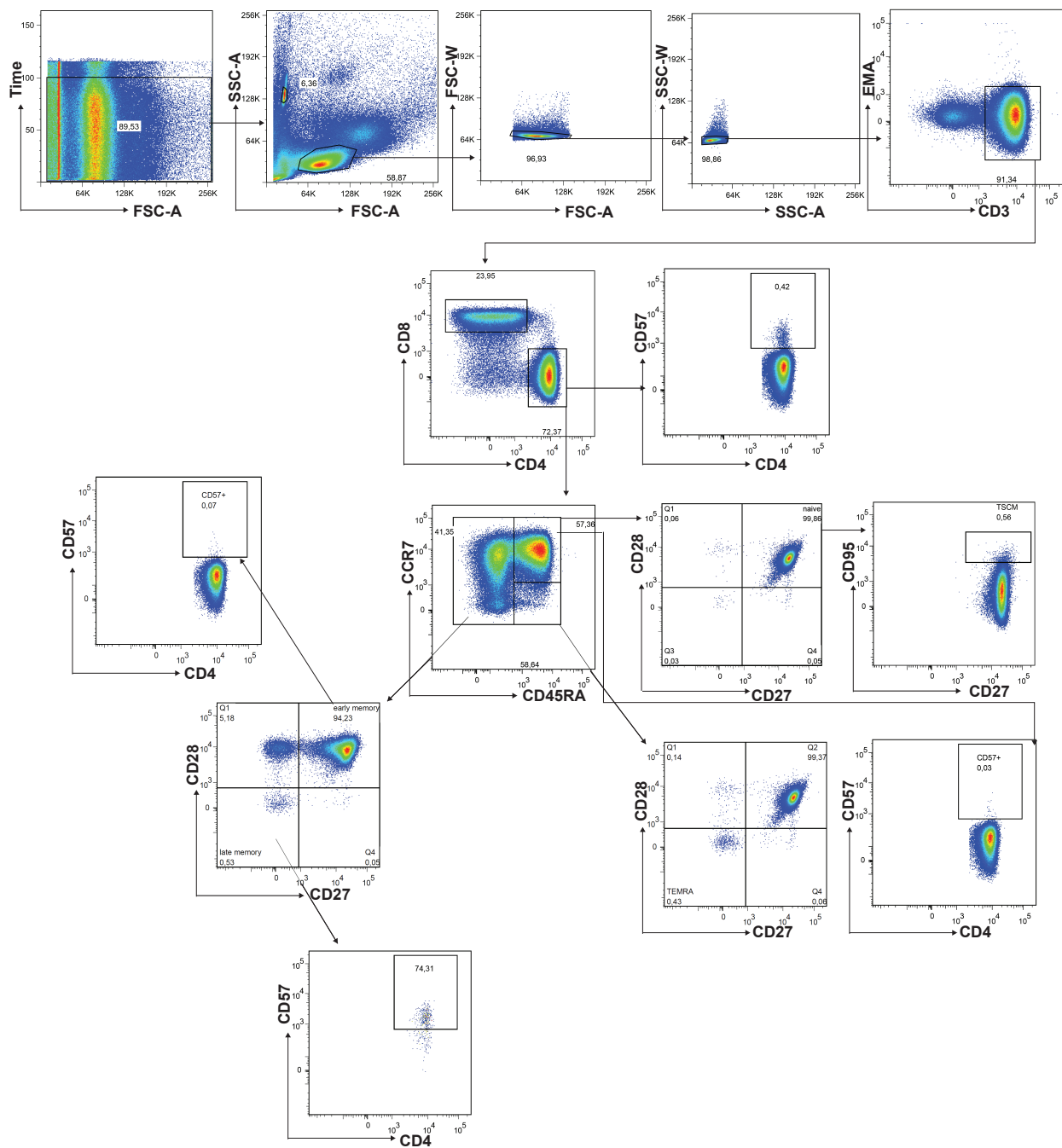

Supplement: Supplementary file 2 — Additional file 2: Supplementary Figure 1. Gating strategy for CD4+ T cells. Supplementary Figure 2 Gating strategy for CD8+ T cells. [file 12979_2020_211_MOESM2_ESM.zip › Supplementary Figure 1_Resubmission.pdf]

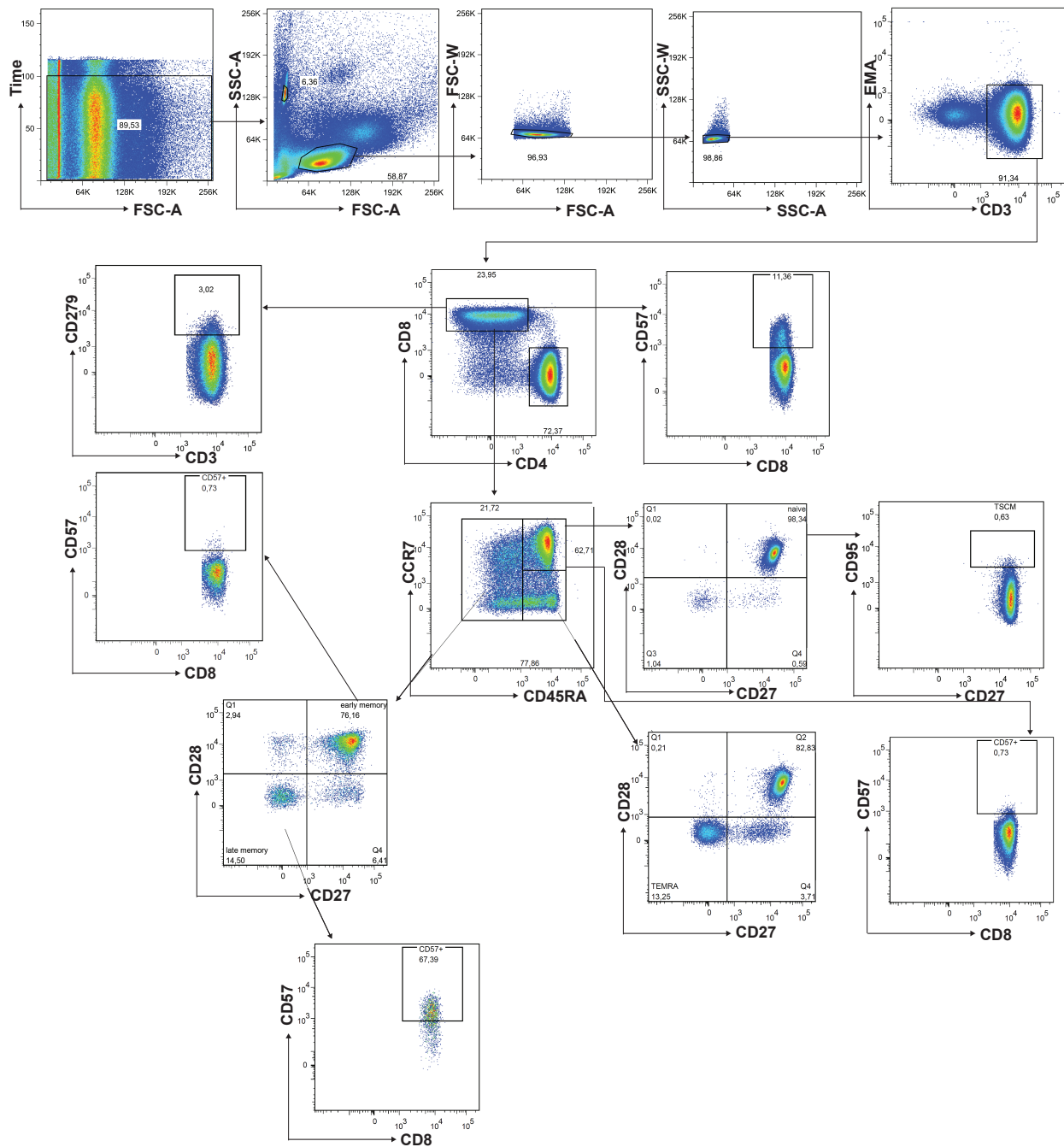

Supplement: Supplementary file 2 — Additional file 2: Supplementary Figure 1. Gating strategy for CD4+ T cells. Supplementary Figure 2 Gating strategy for CD8+ T cells. [file 12979_2020_211_MOESM2_ESM.zip › Supplementary Figure 2_Resubmission.pdf]
